# Scalp acupuncture for post-stroke spastic hemiparesis: A systematic review and meta-analysis

**DOI:** 10.1097/MD.0000000000037167

**Published:** 2024-03-01

**Authors:** Dongxue Zhang, Wei Zou, Baiwen Zhang, Peixin Guo

**Affiliations:** a Heilongjiang University of Chinese Medicine, Harbin, Heilongjiang Province, China; b Acupuncture and Moxibustion Department, First Affiliated Hospital of Heilongjiang University of Chinese Medicine, Harbin, Heilongjiang Province, China; c Key Laboratory of Clinical Molecular Biology of Integrated Traditional Chinese and Western Medicine in Heilongjiang Province, Harbin, Heilongjiang Province, China.

**Keywords:** meta-analysis, scalp acupuncture, spastic paralysis, stroke

## Abstract

**Background:**

Spastic paralysis is one of the most common sequelae of stroke, severely affecting patients’ limb function and reducing their quality of life. Scalp acupuncture (SA) has been shown to significantly improve cerebral blood supply and reduce the severity of limb spasticity. This meta-analysis aims to systematically evaluate the clinical efficacy of SA in the treatment of post-stroke spastic paralysis, providing evidence-based medicine for clinical management of this condition.

**Methods:**

We comprehensively searched databases including China National Knowledge Infrastructure, Wanfang Data, VIP Chinese Science and Technology Periodical Database, China Biomedical Literature Database, PubMed, Embase, and Cochrane Library. Randomized controlled trials investigating the efficacy of SA in post-stroke spastic paralysis were identified until July 28, 2023. Meta-analysis was conducted using RevMan 5.4 and Stata17.0.

**Results:**

A total of 16 studies were included. Meta-analysis showed that the modified Ashworth spasticity assessment scale in the SA group was significantly higher than that in the rehabilitation group (mean difference [*MD*] = −0.56, 95% confidence interval [*CI*] [−0.75, −0.37], *Z* = 5.67, *P* < .00001). The simplified Fugl-Meyer motor function assessment scale in the SA group was significantly higher than that in the rehabilitation group (*MD* = 5.86, 95% *CI* [3.26, 8.46], *Z* = 4.41, *P* < .0001). The modified Barthel index assessment scale in the SA group was significantly higher than that in the rehabilitation group (*MD* = 5.79, 95% *CI* [4.73, 6.84], *Z* = 10.77, *P* < .00001). Additionally, the clinical effective rate in the SA group was significantly higher than that in the rehabilitation group (relative risk = 1.25, 95% *CI* [1.16, 1.36], *Z* = 5.42, *P* < .00001).

**Conclusion:**

SA combined with rehabilitation therapy has certain advantages in reducing limb spasticity, improving limb function, and enhancing activities of daily living in patients with post-stroke spastic paralysis. This study provides reference and theoretical support for the promotion of SA in the treatment of this condition.

## 1. Introduction

Post-stroke spastic paralysis, a major sequelae of stroke, is an abnormal motor pattern primarily characterized by increased muscle tension. It is caused by the reflexive enhancement of spinal motor neurons following damage to the higher motor centers.^[1]^ According to the Global Burden of Disease Study 2019, there were 940,000 new cases of stroke and 760,000 deaths in China over 3 years.^[2]^ Approximately 65% of stroke patients suffer from spastic paralysis of the limbs, with some patients even experiencing long-term muscle spasms.^[3]^ If not treated promptly, this can lead to limb pain, muscle atrophy, joint stiffness, and even deformity, severely reducing the patient’s ability to perform daily activities and impacting their quality of life.^[4]^

Modern medicine posits that the neurons within the central nervous system have the ability to reorganize both structurally and functionally after a stroke, with some neurons able to regenerate under certain conditions, thereby partially restoring function.^[5]^ Acupuncture is a common traditional Chinese medicine treatment for post-stroke limb spasms. Studies have shown that acupuncture can improve cerebral blood supply, promote neuron recovery, and thus alleviate limb spasms.^[6,7]^ A meta-analysis of randomized controlled trials (RCTs) comparing acupuncture to sham acupuncture or standard treatment showed that acupuncture is effective in improving scores on the modified Ashworth spasticity assessment scale (MAS).^[8]^

Scalp acupuncture (SA) is a type of acupuncture technique that combines traditional Chinese meridian theory with modern cortical functional localization, offering greater therapeutic effects in treating cerebral diseases. Research indicates that SA can induce neuronal excitation in brain regions, awakening neurons that are in shock or dormant after a stroke.^[9]^ Combined with rehabilitation training, this can increase the frequency of motor nerve impulses in the cerebral cortex, enhancing its sensitivity and excitability, thereby facilitating the recovery and consolidation of damaged neural functions.^[10]^ Therefore, incorporating SA into the rehabilitation of patients with post-stroke spastic paralysis can effectively increase patient engagement in treatment and accelerate the reconstruction of motor function. Based on this, we have conducted a systematic review to provide evidence-based medical evidence for clinical practice through the analysis of RCT results.

## 2. Materials and methods

As this is a systematic review and meta-analysis of previous research, ethical approval and patient consent are not required.

The present study was a systematic review of meta-analyses based on the Preferred Reporting Items for Systematic Evaluation and Meta-Analyses (PROSPERO). It has been registered in the PROSPERO International Prospective Systematic Evaluation Registry (registration number: CRD42023455579).

### 2.1. Types of studies

RCTs of SA treatment for post-stroke spastic paralysis were included, with no restrictions on age, gender, or case source, provided they met internationally recognized diagnostic standards for the disease.

### 2.2. Interventions

RCTs did not limit the duration or frequency of treatment. The control group received rehabilitation treatment or standard treatment combined with rehabilitation, while the experimental group received SA in addition to the interventions in the control group. Acupuncture points were limited to the scalp, excluding auricular acupuncture, body acupuncture, electroacupuncture, and moxibustion. The acupoints were mostly located in the motor and/or sensory areas of the scalp on the opposite side of the paralyzed limb. The angle between the needle and the scalp was 15–30°. The needle was retained for 0.5–6 hours, with a depth of 2–4 cm. Treatment was administered once a day, 5–7 days a week, for a course of 4 weeks to 3 months.

### 2.3. Outcome indicators

The MAS was used to assess the degree of patient spasticity. The simplified Fugl-Meyer motor function assessment scale (FMA) was used to assess motor function. The modified Barthel index (MBI) was used to assess the patient’s ability to perform daily activities. In addition, the overall clinical effectiveness and adverse events were also included.

### 2.4. Literature retrieval

Literature searches were conducted by 2 researchers. The databases searched included the China National Knowledge Infrastructure, Wanfang Data, VIP Chinese Science and Technology Periodical Database, China Biomedical Literature Database, PubMed, Embase, and the Cochrane Library. The search period was from the inception of each database to July 28, 2023. The search language was Chinese and English. The search was conducted using a combination of subject words and free words. Subject words included “cerebral apoplexy,” “Spastic Hemiplegia,” and “scalp acupuncture.” Free words included “Strokes,” “Cerebrovascular Accident,” “Cerebrovascular Accidents,” “CVA (Cerebrovascular Accident),” “Hemiplegias,” “Hemiplegia, Transient,” “Monoplegia,” “Interactive scalp acupuncture,” etc (Search strategy: Table S1, Supplemental Digital Content, http://links.lww.com/MD/L792).

### 2.5. Literature screening

All retrieved literature was organized using EndNote X9. Two independent evaluators screened the literature that met the inclusion criteria by reading the title and abstract. In case of disagreement, a third researcher was involved in the arbitration. The remaining literature was read in full to make the final decision on inclusion or exclusion.

### 2.6. Data extraction

Data from all the included literature was independently extracted by 2 researchers. This information was cross-checked and standardized. The content included: author, publication time, sample size, baseline, interventions, course, and outcome indicators.

### 2.7. Risk of bias and quality assessment

The risk of bias in the included studies was assessed using the Cochrane Handbook’s recommended bias risk assessment tool. The 7 aspects evaluated were: (1) method of generating random sequence; (2) allocation concealment; (3) blinding of researchers and subjects; (4) blinding of outcome assessors; (5) completeness of outcome data; (6) selective reporting of study results; (7) other biases. For each study, a judgment of “yes” (low risk of bias), “no” (high risk of bias), or “unclear” (lack of relevant information or uncertainty about bias) was made for each item. Two researchers independently completed the assessment for each included study. In case of disagreement, a third researcher was involved in resolving the issue.

### 2.8. Statistic analysis

The statistical software RevMan 5.4 was utilized for the Meta-analysis of the aforementioned outcome indicators. For count data, the relative risk was used as the statistical measure, while for measurement data, the mean difference (*MD*) was used. All effect sizes were represented with a 95% confidence interval (*CI*). Heterogeneity was assessed using the Q test. A fixed-effects model was chosen for analysis when *I*^2^ ≤ 50%, while a random-effects model was used when *I*^2^ > 50%. Additionally, the source of heterogeneity was explored through subgroup analysis or sensitivity analysis. If the influence of heterogeneity was excluded and *I*^2^ ≤ 50%, a fixed-effects model was used for analysis. If the cause of heterogeneity could not be identified, a descriptive analysis was conducted based on the original literature. The funnel plot analysis and Egger test (conducted in Stata 17.0 software) were employed for the assessment of publication bias risk pertaining to outcome measures across ≥10 included studies. If the scatter plot in the funnel plot was roughly symmetrical and the *P* value from Egger test was >.05, it indicated no significant bias and the conclusion was reliable.

## 3. Results

### 3.1. Literature search results

**Figure 1.**
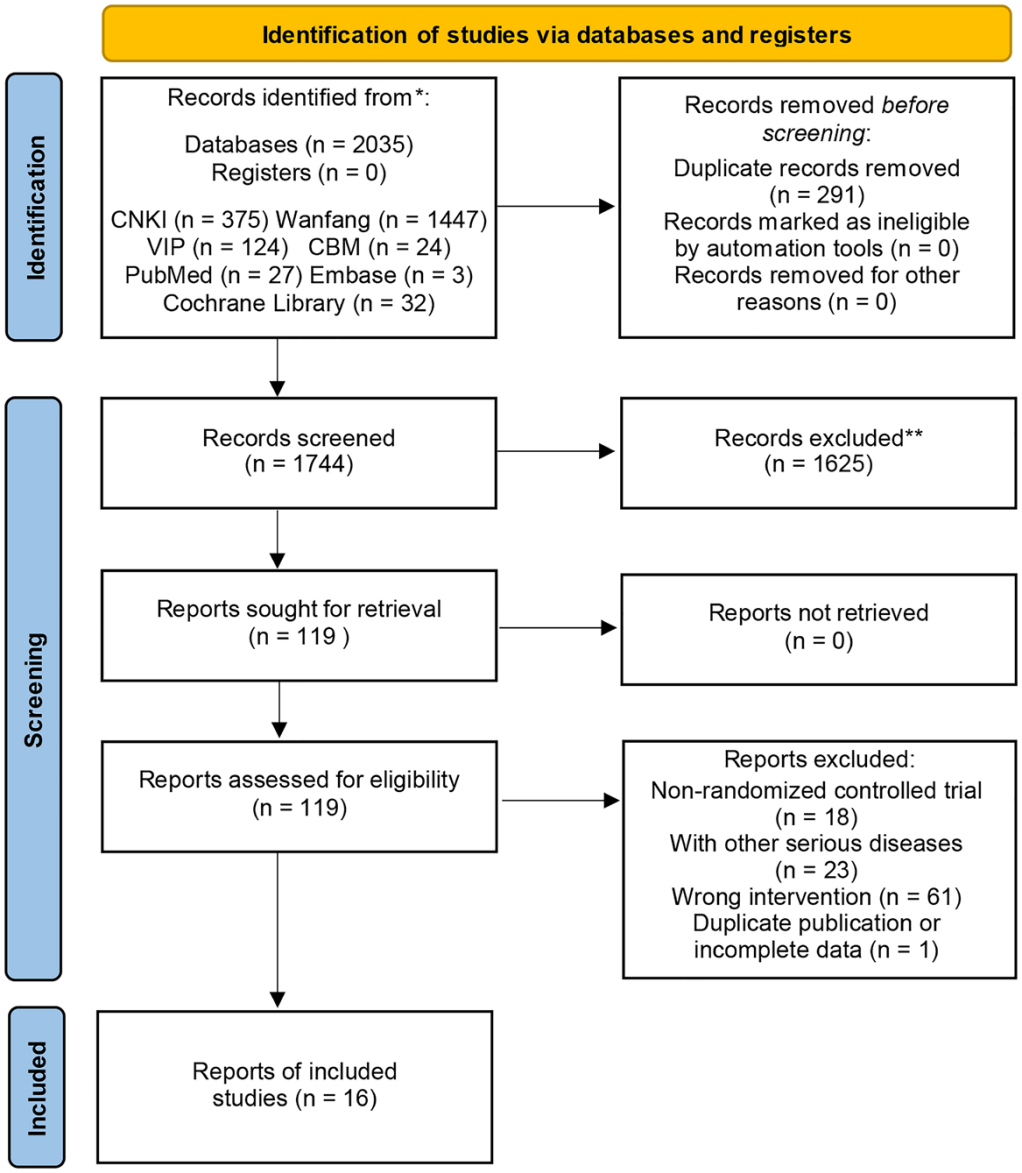
The flowchart of search results for meta-analysis.

A total of 2035 articles were retrieved in this study, among which 291 were duplicate publications. By reading the titles and abstracts of the retrieved articles, 1625 non-RCT studies and studies not relevant to our research topic were excluded. After further reading the full text, 18 RCT studies were collected that used SA as a treatment measure for post-stroke spastic paralysis. During the extraction of outcome indicators, one RCT with incomplete outcome indicators was excluded. One RCT with a short treatment period was also excluded. Finally, 16 RCTs^[11–26]^ were included. All included studies had comparable baselines. The flow chart of literature screening is shown in Figure 1.

### 3.2. Inclusion of literature features

This study included 16 RCT articles, all published between 2013 and 2023, with a total of 1249 patients with post-stroke spastic paralysis (624 in the treatment group and 625 in the control group). The largest sample size was 160, and the smallest was 40. There were 12 RCTs^[11,13–18,21–24,26]^ in the control group with rehabilitation treatment alone. Two^[19,25]^ combined rehabilitation with intelligent physical exercise training, one^[12]^ combined rehabilitation with neuromuscular electrical stimulation, and one^[20]^ combined rehabilitation with music therapy. The treatment group combined SA treatment methods on the basis of the control group’s treatment (Table 1).

**Table 1:** Basic characteristics of the included studies.

Author and year	Sex (M/F)		Age		Treatment duration (d)	Sample size		Intervention		Outcome indicators
	T	C	T	C		T	C	T	C	
Hu DX 2013	23/33		39–75		28	28	28	SA + rehabilitation therapy	Rehabilitation therapy	①
Yang FN 2021	22/14	12/24	63.46 ± 6.85	61.23 ± 6.42	90	36	36	SA + rehabilitation therapy + NMES	Rehabilitation therapy + NMES	①
Zhang FL 2014	37/25	35/26	62.7 ± 5.4	61.8 ± 6.3	30	62	61	SA + rehabilitation therapy	Rehabilitation therapy	②③④
Zhang P 2018	32/28	37/23	53.29 ± 8.21	53.39 ± 8.27	60	60	60	SA + rehabilitation therapy	Rehabilitation therapy	①②③④
Zhang QS 2022	25/11	22/14	35–70		28	36	36	SA + rehabilitation therapy	Rehabilitation therapy	①②③⑤
Ren T 2023	24/7	25/6	48.28 ± 16.78	55.12 ± 10.37	28	31	30	SA + rehabilitation therapy	Rehabilitation therapy	①②③
Zhang QS 2021	21/10	18/12	61.68 ± 2.37	60.47 ± 2.35	28	36	36	SA + rehabilitation therapy	Rehabilitation therapy	①②③
Yang M 2019	19/17	20/16	64.53 ± 7.59	64.08 ± 8.75	28	36	36	SA + rehabilitation therapy	Rehabilitation therapy	①②③④⑤
Zhu ZJ 2019	17/13	19/11	55.63 ± 13.97	51.33 ± 12.44	28	30	30	SA + rehabilitation therapy + IPET	Rehabilitation therapy + IPET	①②
Jia CJ 2017	14/12	15/10	63 ± 11	58 ± 12	28	26	25	SA + rehabilitation therapy + music therapy	Rehabilitation therapy + music therapy	②③⑤
Ma ZY 2019	10/10	9/11	67.2 ± 5.7	65.4 ± 4.8	28	20	20	SA + rehabilitation therapy	Rehabilitation therapy	①②③
Guo Y 2015	24/21	23/22	62. 8 ± 12.4	63. 1 ± 12.2	30	55	55	SA + rehabilitation therapy	Rehabilitation therapy	④
Zhang XY 2019	23/17	21/19	54.49 ± 4.35	52.44 ± 12.13	42	40	40	SA + rehabilitation therapy	Rehabilitation therapy	①②③④
Xu YH 2015	13/7	12/8	46.3 ± 3.7	45.7 ± 4.8	28	20	20	SA + rehabilitation therapy	Rehabilitation therapy	①②③
Zhang CX 2021	47/45	37/41	51 ± 15	54 ± 16	30	78	82	SA + rehabilitation therapy + IPET	Rehabilitation therapy + IPET	①②③
Wang GS 2018	18/12	17/13	60 ± 8	61 ± 8	20	30	30	SA + rehabilitation therapy	Rehabilitation therapy	②③

IPET = intelligent physical exercise training, NMES = neuromuscular electrical stimulation.

### 3.3. Quality evaluation of included literature

**Figure 2.**
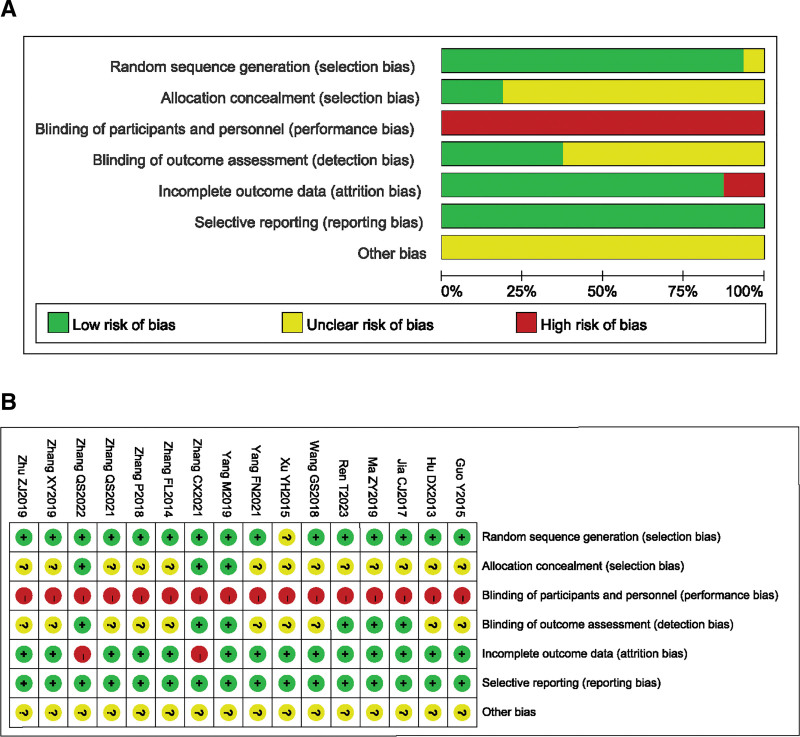
(A) Risk of bias graph. (B) Risk of bias summary.

The included articles were assessed for bias risk using the Cochrane bias risk assessment. Among the 16 included articles, 12 RCTs^[11–14,16,17,19–23,26]^ used random number table method and 3 RCTs^[15,18,25]^ used sealed envelope method. These studies were evaluated as low risk. One RCT^[24]^ only mentioned randomization without describing the specific implementation method and was evaluated as uncertain. Three RCTs^[15,18,25]^ mentioned allocation concealment and were evaluated as low risk. The remaining literature did not mention this. Due to the specificity of acupuncture research, it is difficult to blind the subjects and researchers, so all studies were evaluated as high risk. Six RCTs^[15,16,18,20,21,25]^ mentioned blinding of evaluators and were therefore evaluated as low risk. Two RCTs^[15,25]^ reported dropouts and were evaluated as high risk. All studies did not publish a protocol, so it was impossible to judge whether selective reporting outcomes existed. It was unclear whether other biases existed, so all literature was evaluated as uncertain (Fig. 2A and B).

### 3.4. Meta-analysis results

#### 3.4.1. MAS scores.

**Figure 3.**
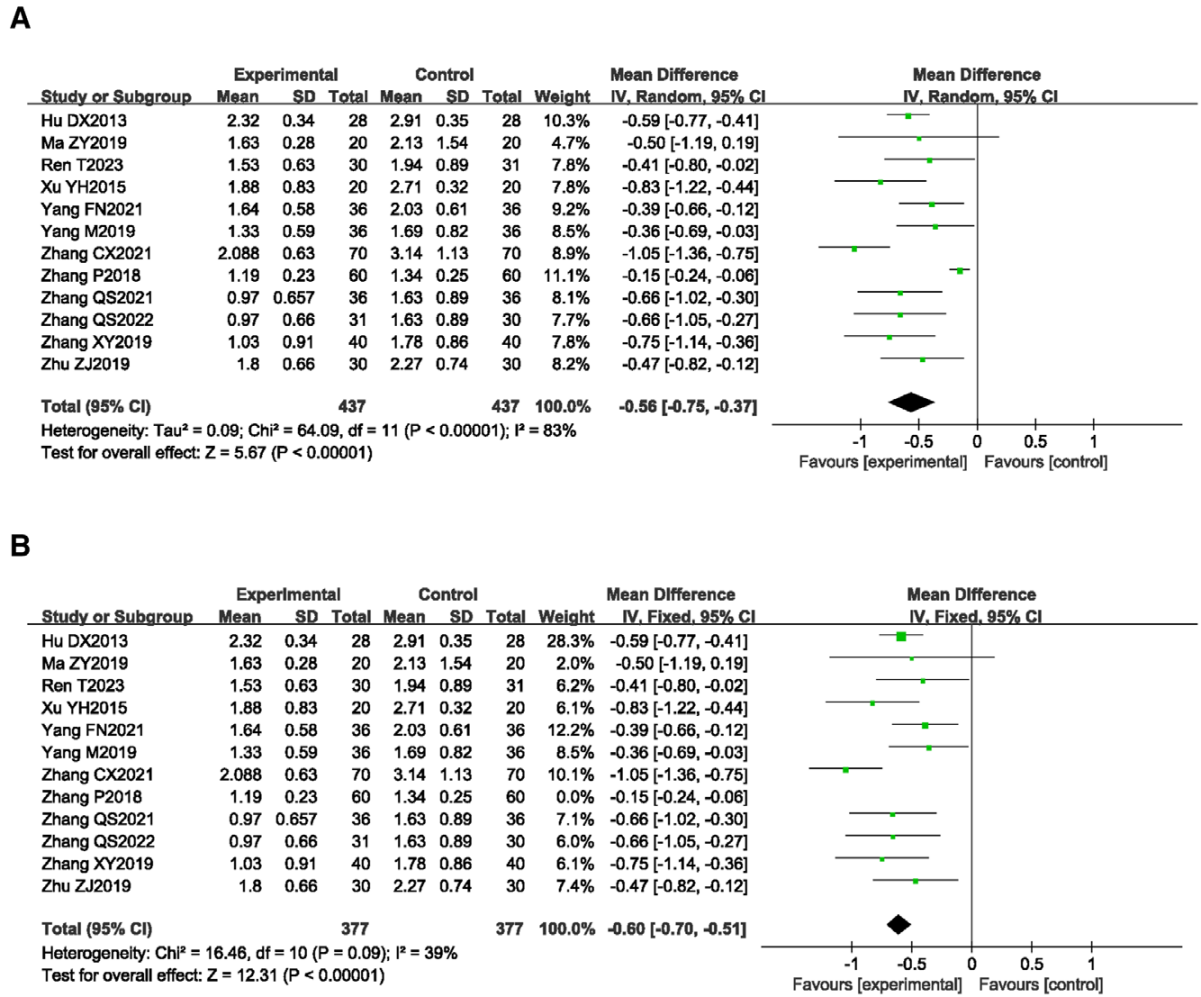
The forest plot of MAS. (A) Meta-analysis results containing MAS for all studies. (B) Sensitivity analysis results for MAS.

A total of 12 studies^[11,12,14–19,21,23–25]^ reported the MAS scores before and after treatment in the SA combined rehabilitation group (treatment group) and the pure rehabilitation group (control group). A total of 874 patients with post-stroke spastic hemiplegia were included in this group, with 437 cases in the treatment group and 437 cases in the control group. The heterogeneity test result was *P* < .00001, *I*^2^ = 83%, indicating significant heterogeneity among the studies. A random effects model was used for the meta-analysis. The meta-analysis result showed that *MD* = −0.56, 95% *CI* [−0.75, −0.37], *Z* = 5.67, *P* < .00001 (Fig. 3A).

Each of the 12 studies was excluded one by one. Sensitivity analysis showed that the heterogeneity significantly decreased after excluding the study by Zhang P 2018. The heterogeneity may be related to the longer retention time of acupuncture (6 hours). The heterogeneity test result was *P* = .09, *I*^2^ = 39%, indicating good homogeneity among the studies, and a fixed effects model was used for the meta-analysis. The meta-analysis result showed that *MD* = −0.60, 95% *CI* [−0.70, −0.51], *Z* = 12.31, *P* < .00001, indicating that SA combined rehabilitation was more effective in improving spasticity in patients with post-stroke hemiplegia than pure rehabilitation, and the difference between the groups was statistically significant (Fig. 3B).

#### 3.4.2. FMA scores.

**Figure 4.**
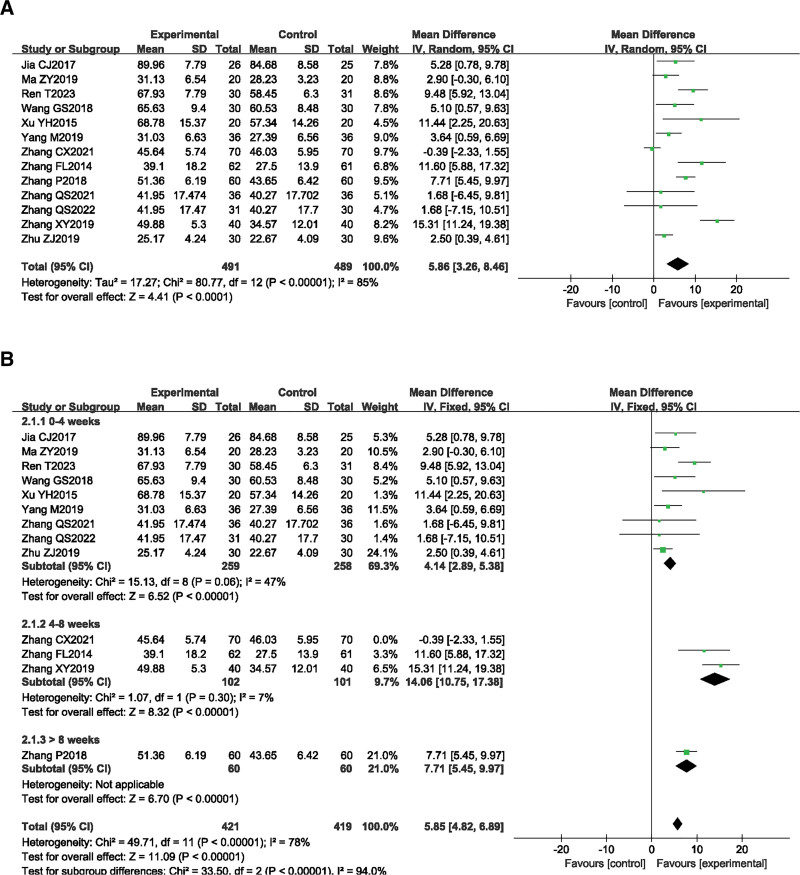
The forest plot of FMA. (A) Meta-analysis results containing FMA for all studies. (B) Results of subgroup analyses of FMA.

A total of 13 studies^[13–21,23–26]^ reported the FMA scores before and after treatment in the SA combined rehabilitation group (treatment group) and the pure rehabilitation group (control group). A total of 980 patients with post-stroke spastic hemiplegia were included in this group, with 491 cases in the treatment group and 489 cases in the control group. The heterogeneity test result was *P* < .00001, *I*^2^ = 85%, indicating significant heterogeneity among the studies, and a random effects model was used for the meta-analysis. The meta-analysis result showed that *MD* = 5.86, 95% *CI* [3.26, 8.46], *Z* = 4.41, *P* < .0001 (Fig. 4A).

Subgroup analysis was conducted based on different treatment periods. There were 9 studies^[15–21,24,26]^ with a treatment period of 0–4 weeks, showing low heterogeneity (*P* = .06, *I*^2^ = 47%). The meta-analysis result showed that *MD* = 4.57, 95% *CI* [2.65, 6.50], *Z* = 4.66, *P* < .00001. There were 3 studies^[13,23,25]^ with a treatment period of 4–8 weeks, showing high heterogeneity (*P* < .00001, *I*^2^ = 96%). Sensitivity analysis was conducted by excluding each literature one by one. After excluding the study by Zhang CX 2021, the heterogeneity significantly decreased (*P* = .30, *I*^2^ = 7%). The heterogeneity may be related to the large sample size of this study (total sample size of 160 cases). The meta-analysis result showed that *MD* = 14.02, 95% *CI* [10.56, 17.48], *Z* = 7.94, *P* < .00001. There was 1 study^[14]^ with a treatment period of more than 8 weeks. This study indicated that the treatment group was significantly superior to the control group in improving patients’ motor function (*P* = .012 < 0.05). The fixed effects model was used to combine the data, and the result suggested that SA combined rehabilitation was more effective in improving motor function in patients with post-stroke spastic hemiplegia than pure rehabilitation (Fig. 4B).

#### 3.4.3. MBI scores.

**Figure 5.**
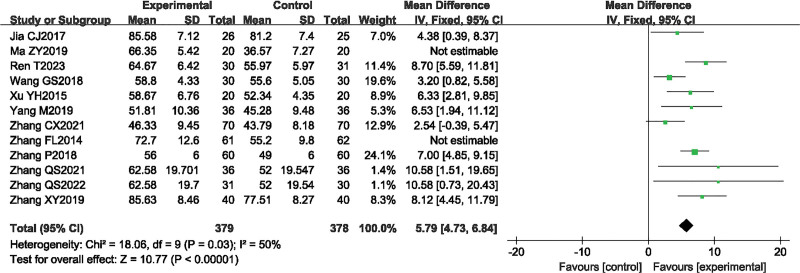
The forest plot of MBI.

A total of 12 studies^[13–18,20,21,23–26]^ reported the MBI scores before and after treatment in the SA combined rehabilitation group (treatment group) and the pure rehabilitation group (control group). A total of 920 patients with post-stroke spastic hemiplegia were included in this group, with 460 cases in the treatment group and 460 cases in the control group. The heterogeneity test result was *P* = .03, *I*^2^ = 50%, indicating good homogeneity among the studies, and a fixed effects model was used for the meta-analysis. The meta-analysis result showed that *MD* = 5.79, 95% *CI* [4.73, 6.84], *Z* = 10.77, *P* < .00001, indicating that SA combined rehabilitation was more effective in restoring daily activities in patients with post-stroke spastic hemiplegia than pure rehabilitation, and the difference between the groups was statistically significant (Fig. 5).

#### 3.4.4. The clinical total effective rate.

**Figure 6.**
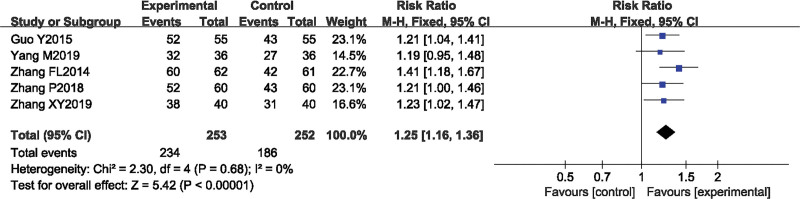
The forest plot of effective rate.

A total of 5 studies^[13,14,18,22,23]^ reported the overall clinical effective rate after treatment in the SA combined rehabilitation group (treatment group) and the pure rehabilitation group (control group). A total of 505 patients with post-stroke spastic hemiplegia were included in this group, with 253 cases in the treatment group and 252 cases in the control group. The heterogeneity test result was *P* = .68, *I*^2^ = 0%, indicating good homogeneity among the studies, and a fixed effects model was used for the meta-analysis. The meta-analysis result showed that relative risk = 1.25, 95% *CI* [1.16, 1.36], *Z* = 5.42, *P* < .00001, indicating that SA combined rehabilitation was more clinically effective in patients with post-stroke spastic hemiplegia than pure rehabilitation, and the difference between the groups was statistically significant (Fig. 6).

#### 3.4.5. Safety evaluation.

Three studies^[15,18,20]^ conducted safety evaluations. Among them, 2 studies^[18,20]^ did not report any adverse events. In one study,^[15]^ 8 people had punctate bleeding at the needle insertion site during the operation, and 2 people had hematoma after needle insertion. After using sterile cotton swabs for compression, no other serious adverse events occurred.

### 3.5. Publication offset test

#### 3.5.1. MAS scale publication bias test.

**Figure 7.**
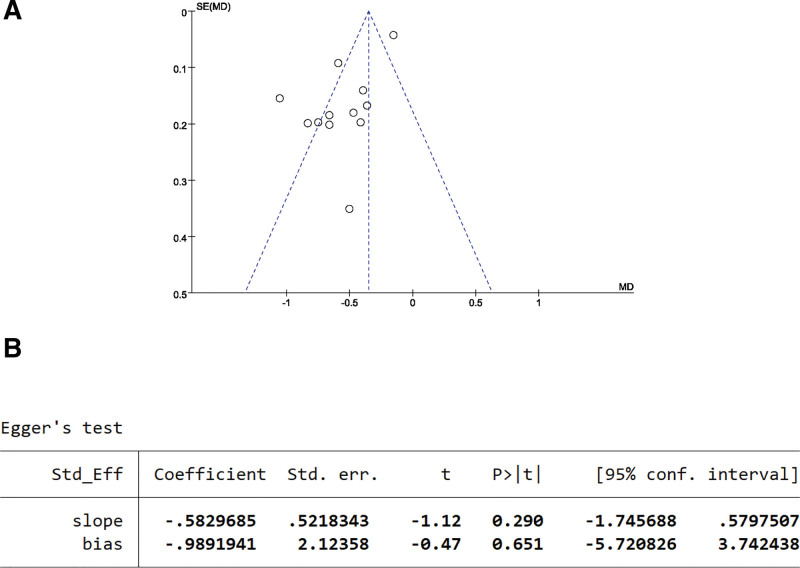
MAS scale publication bias test. (A) The funnel plot of MAS. (B) The Egger test result of MAS.

The publication bias of the 12 studies can be seen from the funnel plot, with most of the scatter points concentrated in the upper-middle part and showing an incomplete symmetrical distribution along the dashed line (Fig. 7A). The Egger test result showed *P* = .651 > 0.05, indicating no publication bias in the literature reporting the MAS scores (Fig. 7B).

#### 3.5.2. FMA scale publication bias test.

**Figure 8.**
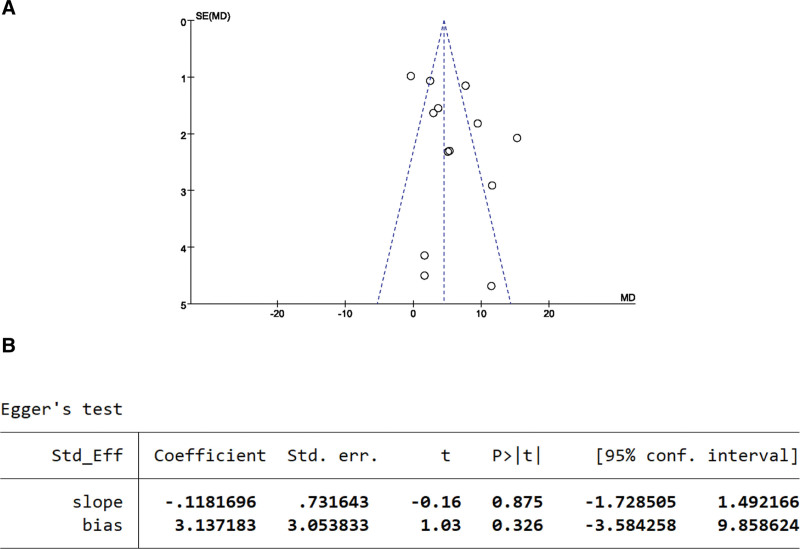
FMA scale publication bias test. (A) The funnel plot of FMA. (B) The Egger test result of FMA.

The publication bias of the 13 studies can be seen from the funnel plot, with the scatter points on the left and right relatively dispersed and roughly symmetrically distributed along the dashed line (Fig. 8A). The Egger test result showed *P* = .326 > .05, indicating no publication bias in the literature reporting the FMA scores (Fig. 8B).

#### 3.5.3. MBI scale publication bias test.

**Figure 9.**
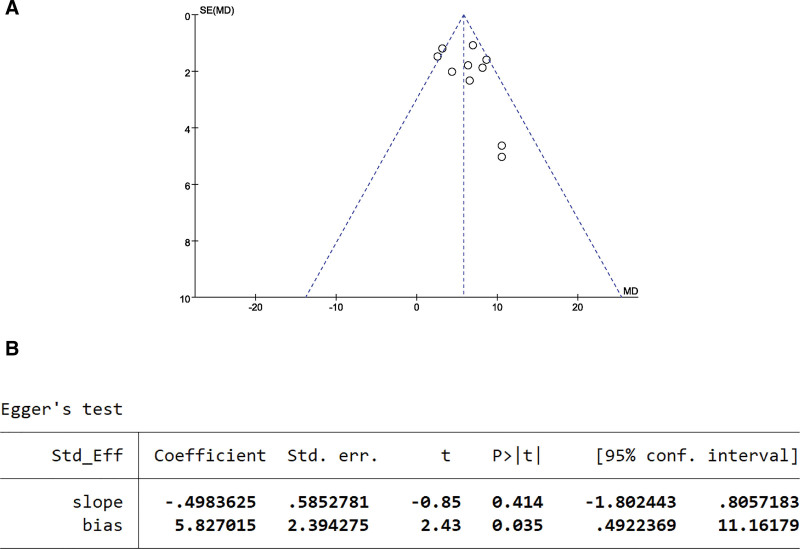
MBI scale publication bias test. (A) The funnel plot of MBI. (B) The Egger test result of MBI.

The publication bias of the 12 studies can be seen from the funnel plot, where the scatter points are relatively dense and concentrated at the top of the funnel, but not symmetrical along the dashed line (Fig. 9A). The result of Egger test showed *P* = .035 < .05, suggesting that there is some publication bias in the literature reporting the MBI scale (Fig. 9B).

## 4. Discussion

In recent years, stroke has become a major cause of death in humans, with most patients developing various complications. Post-stroke spastic paralysis, as one of the severe sequelae of stroke, remains a significant challenge for rehabilitation.^[27]^ Conventional treatments such as drug therapy and rehabilitation therapy have certain effects but are still not ideal. In recent years, an increasing number of studies have shown that acupuncture therapy has significant advantages in treating post-stroke spastic paralysis.^[28–31]^ We conducted a meta-analysis of RCTs on SA treatment for post-stroke spastic paralysis in the hope of providing some reference for clinical decision-making.

This study’s meta-analysis of outcome indicators showed that compared with the control group, SA treatment for post-stroke spastic paralysis had a significant therapeutic effect in improving patients’ spasticity, motor function, and daily living ability. Therefore, combining SA with rehabilitation treatment may be the preferred treatment for post-stroke spastic paralysis and should be given attention in future clinical practice.

The literature included in this study had a wide range of treatment periods, with most trial periods being 4 weeks and the longest trial period being 3 months. In the subgroup analysis of the FMA scale, this factor was used as the basis for grouping. The results showed that the treatment group’s score changes were better than those of the control group, and there were significant differences between different subgroups. Therefore, the treatment period may be one source of heterogeneity in this study. Sensitivity analysis found that differences in acupuncture time and sample size were also causes of heterogeneity. In terms of safety, only one study reported adverse reactions during the trial, namely, pinpoint bleeding or hematoma at the acupuncture site. The treatment method was to apply pressure with a sterile cotton swab.

Influenced by various factors, the quality of the literature included in this study is generally low. The main factor is that the limitations of acupuncture treatment itself make it almost impossible to conduct RCT research in a blinded manner. This factor leads to the generation of placebo effects or selective shifts. Among the included literature, only one study^[25]^ conducted follow-up, and the rest did not mention it. This factor makes it impossible to exclude the immediate effect of acupuncture treatment. In addition, only 3 studies reported adverse reactions during the trial. In terms of intervention measures, the selection of acupoints and the diversity of acupuncture techniques are characteristics of traditional Chinese medicine acupuncture treatment. When treating specific individuals, clinicians need to make judgments based on their professional knowledge. Similarly, MAS, FMA, MBI, and other outcome indicators are subjective judgment indicators, which are easily influenced by the personal clinical experience of clinicians. These subjective factors have become the difficulties of this systematic review study. These factors may be the reasons for the general symmetry of each funnel plot. In addition, due to database resource limitations, only Chinese and English literature were retrieved, which may lead to publication bias.

## 5. Limitations and outlook

This study is a meta-analysis conducted primarily on SA as the main treatment method, and the included literature did not limit the rehabilitation training to a specific type. Therefore, we only restricted SA. The intelligent physical exercise training, neuromuscular electrical stimulation, and music therapy mentioned in the article were also defined as “control groups.” Fortunately, we conducted subgroup analysis and sensitivity analysis on them and found that they were not the main sources of heterogeneity. However, further experiments are needed to determine whether these treatment methods will have a positive or negative impact on the treatment effect of SA. In addition, this study has a wide range of literature inclusion (2013–2023), and the study designs are not completely uniform. There are omissions in the study design of earlier literature, and some literature did not evaluate the location of limb spasticity, which is one of the limitations.

Currently, there are still many methodological limitations in clinical trials of SA for post-stroke spastic hemiplegia, and the research results may be biased. In clinical practice, it is still necessary to conduct well-designed, large-sample, multicenter randomized controlled trials to further validate the effectiveness and advantages of SA treatment. Standardize the clinical efficacy evaluation criteria of MAS, FMA, MBI, and other scales. Pay attention to long-term effects. In addition, further exploration is needed to find the optimal combination and timing of SA and rehabilitation training, promoting the integration of traditional Chinese medicine rehabilitation and modern rehabilitation.

## 6. Conclusion

This study has proven that SA combined with rehabilitation treatment has certain advantages in treating the limb spasticity, limb activity, and daily living ability of patients with post-stroke spastic paralysis, providing a reference and theoretical support for the promotion of SA treatment for post-stroke spastic paralysis. Influenced by the quality and quantity of the included literature, future research should focus on designing more perfect, more comprehensive outcome indicators, multi-center, high-quality RCTs.

## Author contributions

**Funding acquisition:** Wei Zou.

**Writing – original draft:** Dongxue Zhang, Baiwen Zhang, Peixin Guo.

**Writing – review & editing:** Wei Zou.

## Supplementary Material


